# Supplementary figures and images for: Comprehensive phenotyping and transcriptome profiling to study nanotoxicity in C. elegans
Source: PeerJ. 2020 Feb 27;8:e8684. doi: 10.7717/peerj.8684 (PMC7049462; doi:10.7717/peerj.8684)

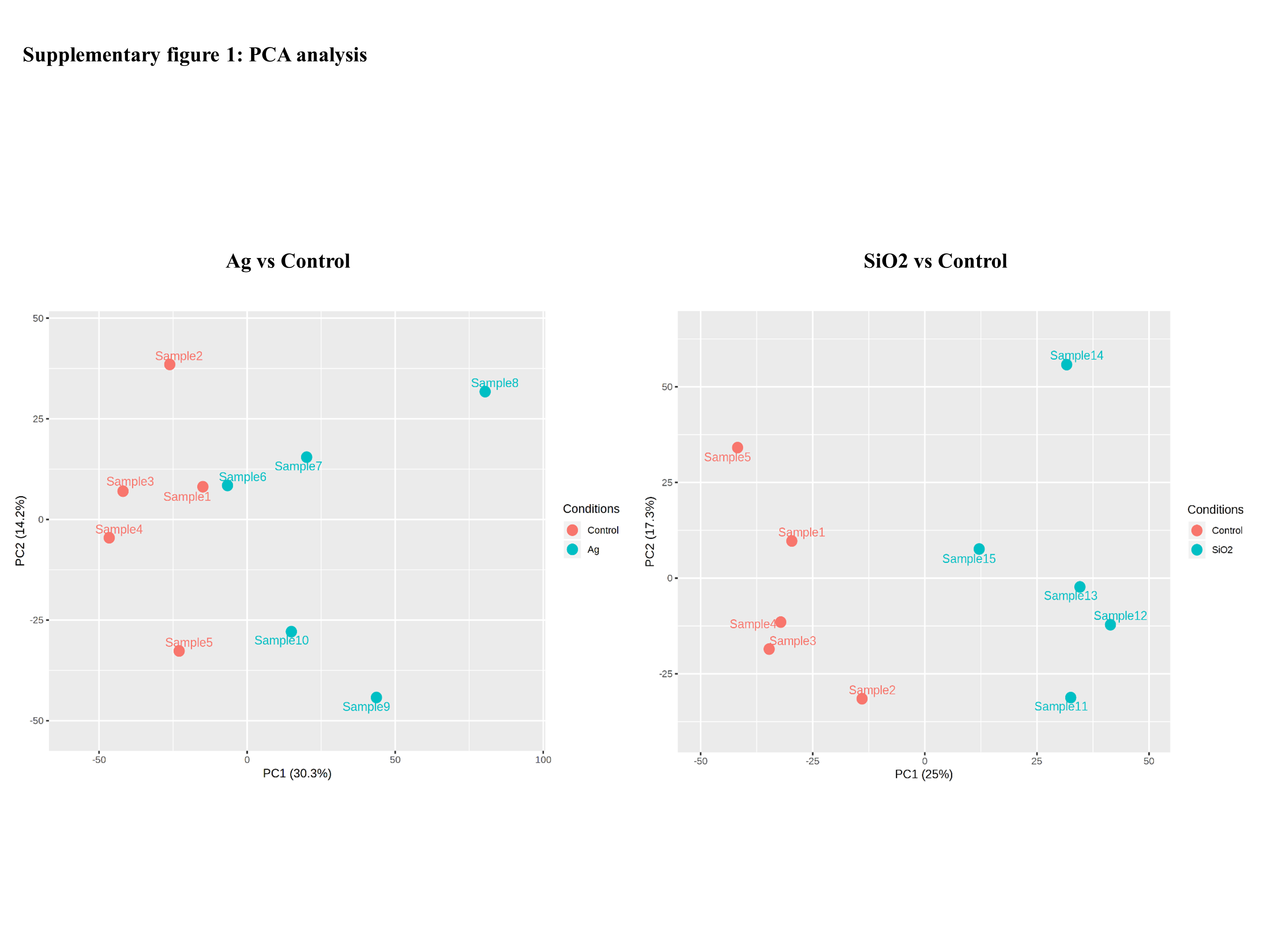

Supplement: Figure S1 — Sample distributionfor each toxicity group (in comparison with control) is shown. [file peerj-08-8684-s001.png]

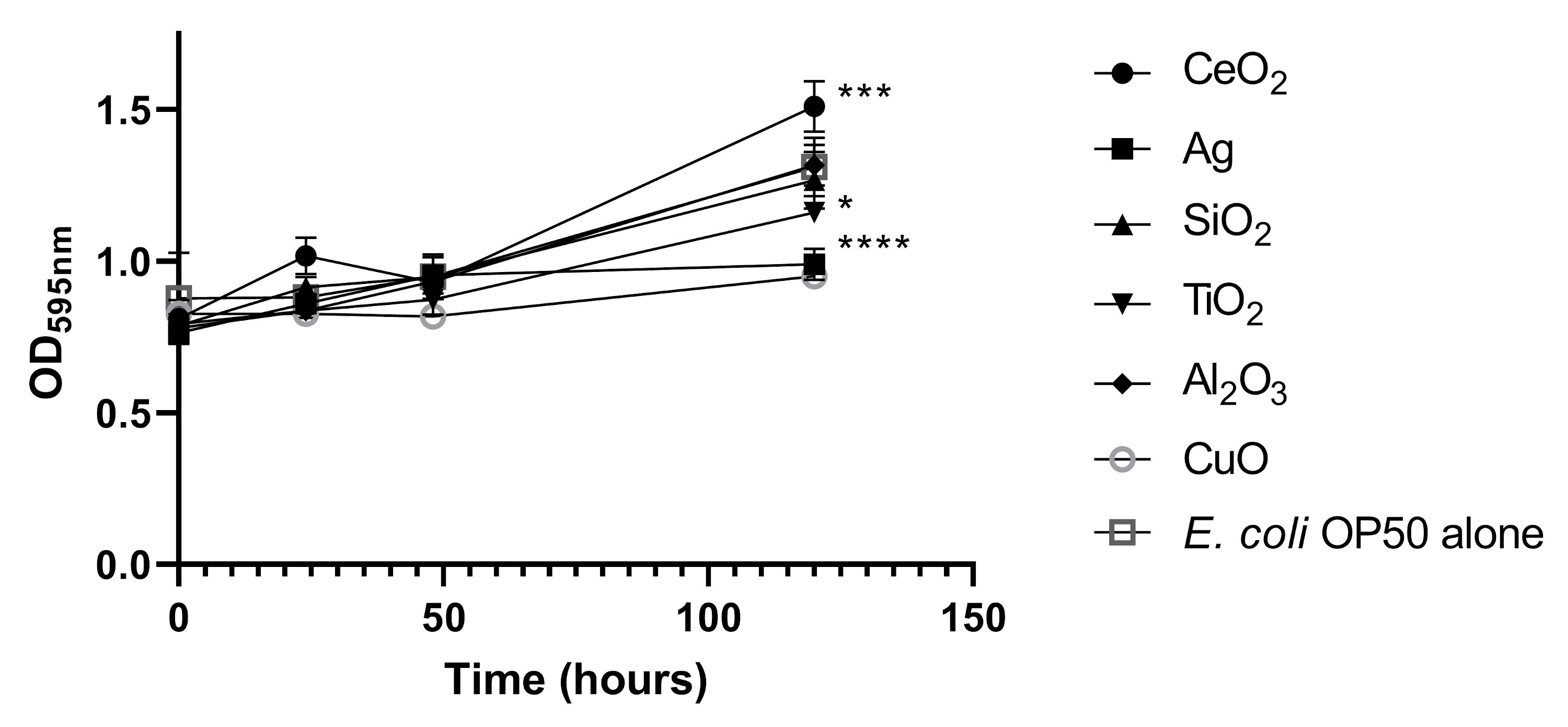

Supplement: Figure S2 — E. coli OP50 was grown in 50 µg/ml of CeO2, Ag, SiO2, TiO2, Al2O3 and CuO nanoparticles in S-medium in a 12-well plate at 21 °C. Bacterial density was measured by taking the OD595 nm values of three independent wells. Statistical significance, after five days incubation, is indicated with an asterisk. * P < 0.05, *** P < 0.01, **** P < 0.0001, compared to E. coli OP50 alone. [file peerj-08-8684-s002.png]
